# Comparison of optical coherence tomography and high frequency ultrasound imaging in mice for the assessment of skin morphology and intradermal volumes

**DOI:** 10.1038/s41598-019-50104-4

**Published:** 2019-09-20

**Authors:** Kornelia Schuetzenberger, Martin Pfister, Alina Messner, Vanessa Froehlich, Gerhard Garhoefer, Christine Hohenadl, Leopold Schmetterer, René M. Werkmeister

**Affiliations:** 1 Center for Medical Physics and Biomedical Engineering, Medical University of Vienna, Währinger Gürtel 18-20, A-1090 Vienna, Austria; 2 Christian Doppler Laboratory for Ocular and Dermal Effects of Thiomers, Medical University of Vienna, Währinger Gürtel 18-20, A-1090 Vienna, Austria; 3 Institute of Applied Physics, Vienna University of Technology, Wiedner Hauptstraße 8-10, A-1040 Vienna, Austria; 4 Department of Biomedical Imaging and Image-guided Therapy, Medical University of Vienna, Währinger Gürtel 18-20, A-1090 Vienna, Austria; 5 Department of Clinical Pharmacology, Medical University of Vienna, Währinger Gürtel 18-20, A-1090 Vienna, Austria; 6 Croma Pharma GmbH, Cromazeile 2, A-2100 Leobendorf, Austria; 7 Singapore Eye Research Institute, 20 College Road Discovery Tower Level 6, The Academia, Singapore, 169856 Singapore; 8 Lee Kong Chian School of Medicine, Nanyang Technological University, 59 Nanyang Dr, Experimental Medicine Building, Singapore, 636921 Singapore

**Keywords:** Preclinical research, Imaging and sensing, Experimental models of disease

## Abstract

Optical coherence tomography (OCT) and high-frequency ultrasound (HFUS), two established imaging modalities in the field of dermatology, were evaluated and compared regarding their applicability for visualization of skin tissue morphology and quantification of murine intradermal structures. The accuracy and reproducibility of both methods were assessed *ex vivo* and *in vivo* using a standardized model for intradermal volumes based on injected soft tissue fillers. OCT revealed greater detail in skin morphology, allowing for detection of single layers due to the superior resolution. Volumetric data measured by OCT (7.9 ± 0.3 μl) and HFUS (7.7 ± 0.5 μl) were in good agreement and revealed a high accuracy when compared to the injected volume of 7.98 ± 0.8 µl. *In vivo*, OCT provided a higher precision (relative SD: 26% OCT vs. 42% HFUS) for the quantification of intradermal structures, whereas HFUS offered increased penetration depth enabling the visualization of deeper structures. A combination of both imaging technologies might be valuable for tumor assessments or other dermal pathologies in clinical settings.

## Introduction

Routine physical examination and diagnostics in dermatology are commonly performed using simple optical techniques such as the use of a dermatoscope or a magnifying glass, or the analysis of photographs of skin^1^. These operator-dependent techniques require an experienced investigator and only allow for observation of the skin surface without accessing deeper structures. For the diagnosis of various cutaneous pathologies, morphological features in deeper layers of the skin need to be assessed. Thus, excisional biopsies of the region of interest are often required, accompanied by increased risk of infection, scarring skin, and delayed wound healing^2–4^.

New imaging modalities capable of visualizing deeper structures within the skin and adnexa non-invasively would be beneficial for both clinical diagnosis and research^5^. In addition, imaging approaches that generate quantitative and functional datasets while being less operator-dependent are worth pursuing.

Potential imaging modalities include confocal laser microscopy (CLM)^2^, epiluminescence microscopy (ELM)^6,7^, high-frequency ultrasound (HFUS)^8,9^, multiphoton tomography (MPT)^10^, magnetic resonance imaging (MRI)^11,12^ and optical coherence tomography (OCT)^1,13,14^. Some of these modalities provide either very high resolution but only of the superficial layers (e.g. ELM) or a favorable penetration depth but only at a coarse resolution (e.g. MRI). OCT and HFUS provide the most favorable balance between spatial resolution and imaging depth. With a spatial resolution ranging from a few micrometers to tens of micrometers and an imaging depth of several millimeters, these technologies are ideally suited for dermal imaging in preclinical and clinical settings and for the investigation of various skin pathologies.

OCT is a non-invasive, contact-free and rapid imaging modality, providing almost cellular resolution and offering the possibility to acquire both cross-sectional images and three-dimensional data sets of the skin and its microstructure. Depending on the spectral bandwidth of the light source, typical systems provide an axial resolution in the micrometer range and achieve a penetration depth of around 1 to 2 mm, particularly when using light sources in the 1300 nm range. OCT has been employed to investigate a variety of skin pathologies and was shown to provide additional information about structural and cellular alterations^15^. In psoriasis-like inflammation in a murine model, OCT was capable of detecting changes in dermal reflectivity with progressing severity^16^. Furthermore, the technology showed significant correlation with histological grading of biopsies of murine and human skin^16,17^.

HFUS is an imaging method that relies on the detection of pulse-echoes from acoustic waves emitted and detected by transducers. Different tissue types exhibit characteristic acoustic impedances, generating contrast in the cross-sectional images (B-scans). The use of higher frequencies leads to a higher resolution, but at the cost of decreased penetration depth. In dermatology, high-frequency transducers with a frequency range from 10 to 100 MHz are commonly utilized to obtain cross-sectional images of the skin and its microstructure^18,19^. Applications of HFUS in dermatology include surveys of tumor and inflammatory diseases, epidermal thickness measurements, evaluation of ultrasound-mediated drug delivery and proposed treatments^20,21^. In a preclinical study, measuring skin involvement in different models of systemic sclerosis in mice, a 50 MHz transducer achieved axial and lateral resolutions smaller than 100 µm with a depth of field of 1.4 mm and enabled visualization of multiple skin layers^22^.

There is an increasing clinical interest for accurate and non-invasive skin imaging techniques, especially regarding the continuous improvement in resolution. Additionally, functional imaging allows for new applications such as label-free visualization of blood flow using inherent contrast mechanisms.

OCT and HFUS have been used to investigate the spatial extent, depth and exact location of intradermal volumes and lesions such as in bullous diseases^23–25^, soft tissue filler augmentations^26,27^ and acne vulgaris^28,29^. Furthermore, HFUS and OCT have been applied in experimental studies to evaluate the accuracy and reproducibility of pre-surgical depth assessments of cancerous skin lesions^19,30^.

In the present study, we employed OCT and HFUS for real time non-invasive imaging in an *in vivo* murine model for visualization of skin morphology and determination of intradermal volumes. The primary aim was to compare accuracy and reproducibility of intradermal volume measurements between both methods, employing a standardized animal model and predefined injection volumes. Furthermore, data was compared in terms of penetration depth, resolution and contrast. Anatomical landmarks in cross-sectional images of both modalities were correlated with histology. The applicability and diagnostic potential of both methods in preclinical studies is discussed. To the best of our knowledge, this is the first study analyzing the capabilities of OCT and HFUS in both visualization of skin morphology and quantification of standardized intradermal volumes in a murine model.

## Results

**Figure 1:**
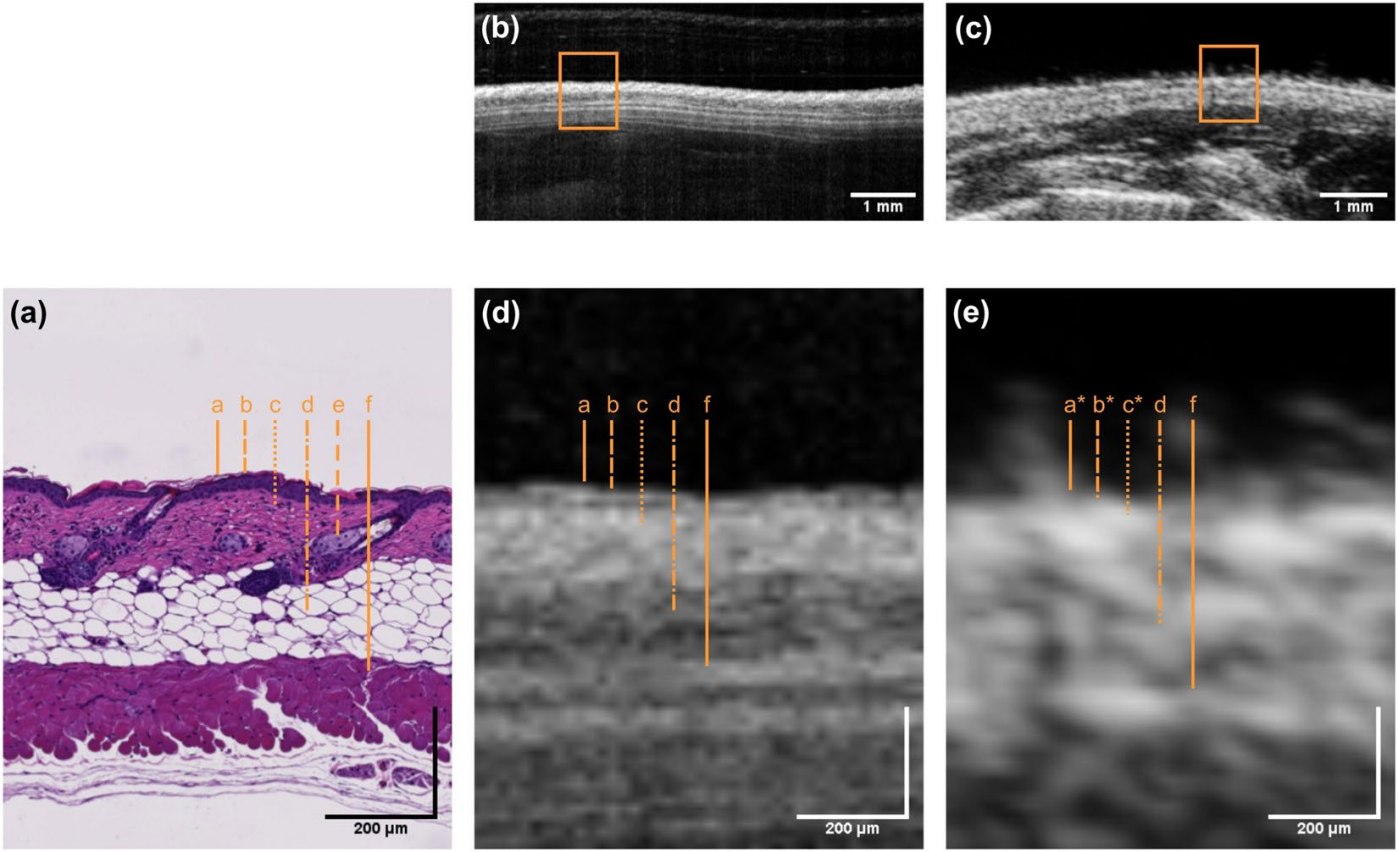
Exemplary cross-sectional images of murine skin obtained by (**b**) OCT and (**c**) HFUS and corresponding (**a**) histology (H&E staining). Enlarged cross-sections as indicated by the orange rectangles are depicted in (**d**,**e**). Dashed orange lines in image (**a**,**d**,**e**) indicate the skin layers corresponding to: (**a**) epidermis, (**b**) papillary dermis, (**c**) reticular dermis, (**d**) subcutis, (**e**) sebaceous glands and hair follicles and (**f**) muscle. Skin layers with letters labeled with an asterisk are not distinguishable.

Examples of cross-sectional images of murine skin obtained by OCT and HFUS and the corresponding histological images are depicted in Fig. 1. The high-resolution OCT system facilitates the discrimination of distinct skin layers: epidermis, papillary dermis and reticular dermis. The penetration depth of the OCT system of more than 1 mm also allows for visualization of the exterior part of the subcutis. Skin thickness measurements performed on OCT B-scans revealed a full skin thickness of 500.2 ± 44.2 μm. The scans also allowed to measure the thickness of single layers resulting in the following mean values: combined epidermis and papillary dermis 59.0 ± 15.5 μm; reticular dermis 207.4 ± 25.6 μm; and 233.8 ± 29.7 μm for the subcutis.

Although HFUS is able to display deeper skin structures and offers better contrast, the technology’s axial resolution of 30 µm allows only for the differentiation of dermis and subcutis, but not for a precise assessment of thickness. A full skin thickness measurement performed on HFUS B-scans revealed a mean value of 501.6 ± 69.8 μm.

**Figure 2:**
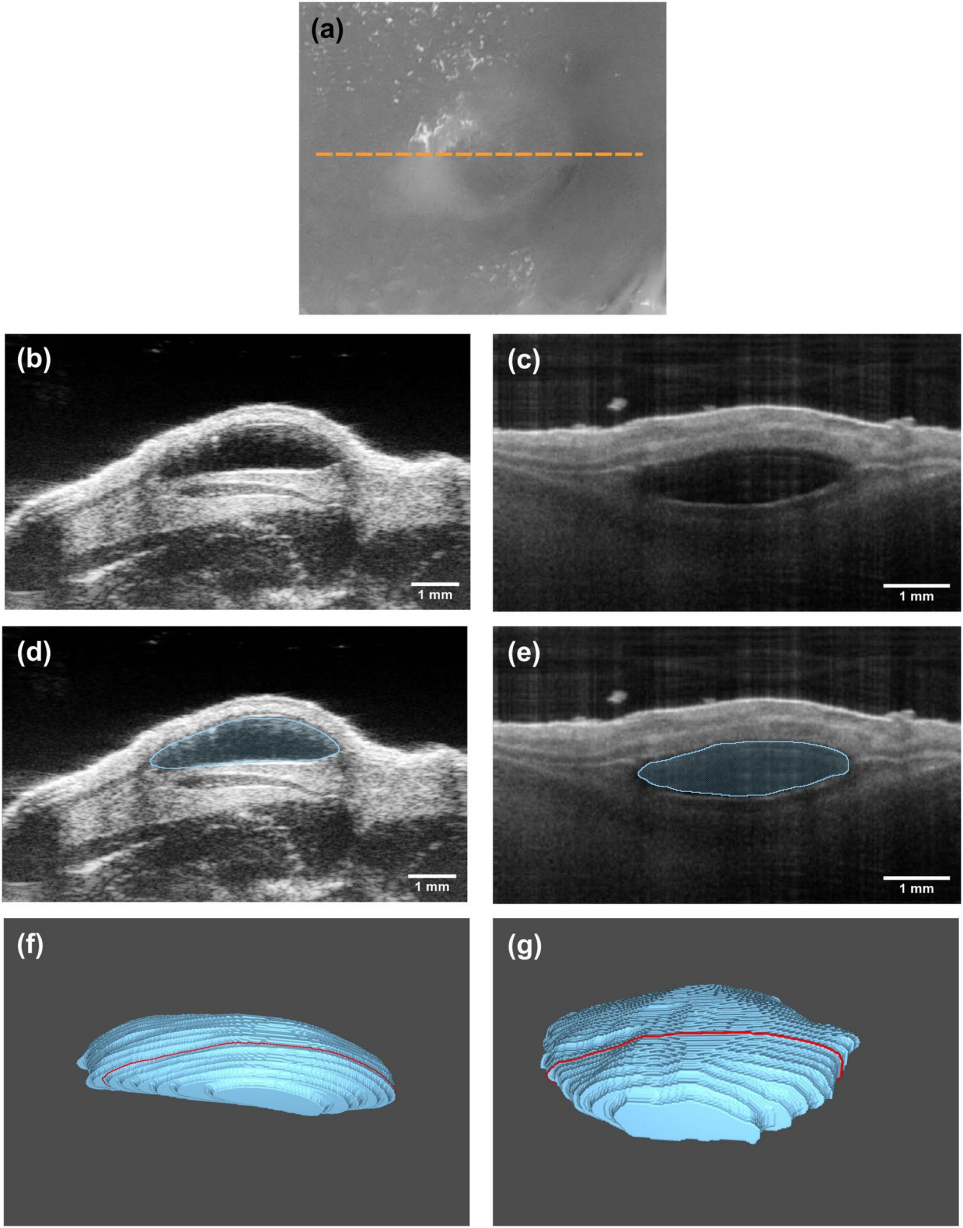
*In vivo* imaging of the murine soft tissue filler model, showing the same filler deposit imaged with OCT and HFUS. (**a**) Photograph of the dorsum of a mouse containing the soft tissue filler. The yellow line indicates the position of the cross-sectional image obtained by (**b**) HFUS and (**c**) OCT. The images of (**d**) HFUS and (**e**) OCT show examples of results of the automatic segmentation algorithm used for calculation of the volumes. Images (**f**,**g**) show 3D representation of intradermal deposit volumes as obtained from HFUS and OCT scanning data, respectively.

Examples of images of intradermal volumes recorded by HFUS and OCT are presented in Fig. 2. Here, representations of raw cross-sectional images, images with automatically segmented filler borders and three-dimensional filler reconstructions are shown. In OCT images, the injected filler deposit presents as homogeneous and hyporeflective due to the absence of distinct scatterers within the volume. Ultrasound images displayed the intradermal deposits as well-defined, hypoechogenic structures with a homogeneous appearance compared to the surrounding tissue.

*Ex vivo* microscopy measurements of the glass sphere revealed a volume of 0.55 ± 0.05 μl. Assessment of the volume based on OCT measurements of the excised skin yielded a mean volume of 0.60 ± 0.06 μl, in good agreement to the actual volume as assessed *ex vivo*. Due to strong scattering artifacts at the borders of the glass sphere, determination of its volume *ex vivo* via HFUS was not possible.

Weighing the excised skin samples immediately pre and post application of the filler and considering the density of the material, the mean volume was estimated to be 7.97 ± 0.70 μl, which is distinctly lower than the syringe injection volume of 10 μl. The mean volume detected by OCT was 7.88 ± 0.29 μl and the mean volume determined by HFUS was 7.73 ± 0.53 μl, all differences being not statistically significant (OCT vs. weight: p = 0.28; HFUS vs. weight: p = 0.34; OCT vs. HFUS: p = 0.64).

**Figure 3:**
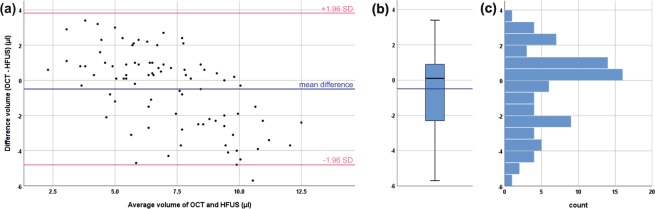
*In vivo* volumetric assessment of soft tissue fillers. (**a**) Bland-Altman plot, (**b**) box plot and (**c**) histogram comparing data obtained with OCT and HFUS.

*In vivo* assessment of the intradermal tissue fillers yielded mean volumes of 6.9 ± 1.8 μl and 7.3 ± 3.1 μl (n = 84) for OCT and HFUS measurements, respectively (statistically significant difference; p = 0.043). A strong correlation was found between values detected with OCT and values detected with HFUS (r = 0.707; p < 0.001). Figure 3(a) analyzes the acquired data using a Bland-Altman plot. It can be observed that the deviation between the two methods depends on the absolute value of the measured volume. When assessing smaller deposits, OCT measurements tend to be larger than HFUS, while the contrary is true for larger deposits. For the latter case, however, the difference between the two methods is considerably larger, as can be seen from the skewed distribution in the histogram (Fig. 3(c)) and the deviation between median and mean difference (box plot, Fig. 3(b)).

## Discussion

Both OCT and HFUS facilitate visualization of skin morphology and three-dimensional *ex vivo* and *in vivo* assessment of intradermal volumes. The contrast in OCT images is given by differences in the refractive indices, the distinct light scattering and absorption properties of the probed tissue. This makes the identification of the distinct skin layers and visualization of morphological features possible. Therefore, possible pathologies of outer skin layers such as non-melanoma skin cancer, benign growths and bullous diseases can be detected via OCT with high resolution.

The contrast in HFUS images, on the other hand, is given by differences in acoustic impedances of the tissue. Lately, transducers using ultrasound frequencies of 100 MHz have been evaluated for skin imaging and shown to provide considerably improved resolutions of 39 µm and 210 µm in axial and lateral directions, respectively, compared to 50 MHz scanners. In the current study, the axial resolution of the custom-built OCT system was about three times higher than that of the employed commercially available HFUS system. This superior resolution revealed greater details of skin morphology and allowed differentiation of more individual skin layers. Thus, the cross-sectional image depicted in Fig. 1(d) allows a clear differentiation of epidermis, papillary dermis, reticular dermis and subcutis and their respective extensions. In contrast, the more limited resolution of the HFUS resulted in an increased appearance of scatterers and blurring of boundaries, thereby hampering layer identification and distinct thickness measurements.

Varkentin *et al*. conducted a comparative study of pre-surgical measurements of skin infiltration depths of tumors and reported a good agreement between histopathology and OCT or HFUS, respectively^19^. On average, OCT slightly overestimated the infiltration depth while assessment via ultrasound resulted in a slight underestimation with a larger absolute deviation. Our preliminary OCT measurements on a glass sphere revealed a very good agreement between the dimensions determined with a microscope and the volume calculated from three-dimensional OCT data. The minor deviation between the two values has to be attributed to the OCT measurement principle: The refraction of the probe beam at oblique interfaces, where a refractive index change occurs, ultimately leads to a slight distortion of the image. Contrary to the glass sphere, the refractive index of both soft tissue fillers and skin tumors is very similar to the surrounding tissue, rendering this effect negligible for the intended application.

The intradermal filler volume, estimated by considering the weight difference of the skin before and after injection, was approximately 20 percent smaller than the syringe injection volume. This significant difference can be attributed to the high murine skin turgor, which led to a partial ejection of the volume directly after the injection. Furthermore, the high viscosity of the filler might result in slight inaccuracies during application. The assessment of intradermal volumes in excised skin *ex vivo* via both imaging methods revealed that volumes assessed with OCT and HFUS were in very good agreement with the injected volume that was estimated from weighing the skin samples.

When measuring the intradermal volume *in vivo*, both methods, again, revealed volumes significantly smaller than the syringe injection volume. The discrepancy can be mainly attributed to the skin turgor leading to partial loss of filler at the injection site. Comparing the results of OCT and HFUS, a noticeably higher standard deviation was observed for the latter. This can be explained by the physical principles of the imaging methods. OCT is based on the detection of backscattered light and, thus, does not require contact between the imaging unit and the probed tissue. In contrast, the ultrasound probe needs to be in contact with the skin via the ultrasound gel. For volumetric imaging, the ultrasound transducer was moved along the skin surface using a linear translation stage. This mechanical motion of the measurement head might have caused an unintended movement of the filler and thus an imperfect sampling of the deposit. The effect was observed to be stronger for larger filler volumes that revealed a stronger bulging of the skin with higher mobility of the deposit, which was indicated by the larger deviation of the two methods for larger deposit sizes (ref. Fig. 3). This, in consequence, hampered precise assessment of intradermal volumes, leading to the observed higher variances of the HFUS results (relative SD: OCT 26%; HFUS 42%). Measurements of intradermal volumes via OCT, on the other hand, do not require any mechanical manipulation of the sample, thus allowing contactless assessment of three-dimensional data sets and, therefore, higher precision as indicated by the smaller standard deviation.

Comparison of the cross-sectional images in Fig. 1(b,c) reveals the superior penetration depth of HFUS over OCT. The penetration depth in OCT is mainly influenced by two factors. The infrared light absorption of water increases with increasing wavelength; restricting the applicability of the 1300 nm wavelength range for imaging of the eye, which mainly consists of water, but favoring this range for assessment of skin, where the water content is only 65 to 75 percent. Furthermore, in this wavelength region, scattering of light in biological tissues decreases monotonically with increasing wavelength, leading to a higher tissue penetration depth of the light source employed in our study. With the current setting, the maximum penetration depth was in the range of two millimeters, allowing quantitative evaluation of superficial alterations of the skin but preventing assessment of pathologies in deeper skin layers.

The attenuation in tissue and, thus, the penetration depth of US is approximately linearly proportional to the frequency. At 50 MHz, this depth is limited to 8 to 9 mm, significantly higher than OCT. In OCT, resolution and penetration depth are not as strongly linked as in ultrasound. The spectral bandwidth of the light source and the specifications of the optical system define the axial and lateral resolutions in OCT, respectively, while the penetration depth is dependent on the aforementioned scattering and water absorption. These are, as explained above, influenced by the central wavelength of the light source. In contrast, for US, the axial resolution is determined by the pulse duration or bandwidth and thus directly linked to transducer frequency and inversely proportional to the penetration depth. Consequently, high-frequency transducers, such as the one employed in the current study, offer a smaller penetration depth than standard US systems typically using frequencies from 2 to 15 MHz. However, despite a resolution that is inferior to OCT, HFUS revealed certain details of the morphology in the upper skin of mice, and allowed visualization of deeper tissues.

While the current study aimed to investigate the characteristics of OCT and HFUS for visualization of skin morphology and measurements of intradermal volumes, functional extensions of both modalities, like Doppler OCT and OCT angiography or Doppler US, can provide additional information about blood flow and perfusion in the probed tissue region and are able to present abnormal vessel formation providing additional diagnostic parameters.

In conclusion, both imaging modalities, OCT and HFUS, allowed for the assessment of skin morphology and determination of intradermal volumes with high accuracy and, therefore, are suited for the investigation of the skin and occurring pathologies in both preclinical and clinical settings. While the major advantage of HFUS lies in the superior penetration depth, allowing assessment of deeper structures, the superior resolution of OCT reveals finer morphological details and provides a higher precision for the determination of geometrical dimensions. For the assessment of skin lesions, a combination of both methods – HFUS for the evaluation of pathological changes in deeper tissue regions and OCT for providing insights into microstructural changes and precise size assessments – could be beneficial for the clinician’s decision about future therapeutic steps and for setting excision margins for tumors.

## Material and Methods

### High-frequency ultrasound

The sonographic evaluation was conducted using a high-resolution ultrasound device (Vevo2100, FUJIFILM VisualSonics Inc.) and a high-frequency transducer probe (VisualSonics MS700, FUJIFILM VisualSonics Inc.). The transducer probe with a frequency of 50 MHz provided a characteristic resolution of 30 µm and a maximum imaging depth of 9.7 mm. For acquisition of volumetric data sets, the transducer was connected to a two-axis translation stage (VisualSonics, FUJIFILM VisualSonics Inc.) with a travel range of 10 mm and a 3D step size of 32 nm. The recording time for one volume was about 30 seconds.

### Optical coherence tomography

Optical coherence tomography data was acquired using a custom-built swept-source OCT system based on an akinetic light source (Insight Photonic Solutions, Inc.) operating at a central wavelength of 1310 nm and providing a spectral bandwidth of 87 nm. Details of the experimental setup and data processing are described elsewhere^31^. For assessment of intradermal volumes, volumetric data sets of a skin area of 10 × 10 mm², each comprising 512 × 1500 × 1536 voxels, were recorded. The OCT system offered resolutions of 6.5 µm and 22 µm in axial and lateral directions in tissue, respectively. The acquisition of one volumetric data set took 14 seconds. The incident power of the probe beam onto the tissue was set to 33 mW, which is well below the safety limit set by the International Electrotechnical Commission^32^.

### *Ex vivo* experiments

To evaluate the accuracy of volumetric measurements acquired with OCT and HFUS, two *ex vivo* experiments were conducted. First, the size of a precision glass sphere (HSI-Solutions GmbH) with a diameter of 1 ± 0.02 mm, a circularity of ≤0.02 mm and a mean volume of 0.52 μl as specified by the manufacturer was assessed with a microscope (Olympus BX51, UPLFLN objective, Olympus Life Sciences) at 4 x magnification and using a scale in the imaging plane. Thereafter, the sphere was imaged five times after insertion into excised murine skin. In the second experiment, a commercially available soft tissue filler (Princess VOLUME, Croma Pharma GmbH), modeling the geometrical proportions of a tumor, was injected into the mouse skin and measured with both modalities. The filler material, a hydrogel with a refractive index of 1.37 at the center wavelength of the light source, consists of chemically cross-linked hyaluronic acid.

Preliminary *in vitro* measurements were conducted to investigate the behavior and detectability of the filler in murine skin. For this purpose, excised murine skin (n = 5) was weighed pre and post application of 10 μl of filler. Finally, intradermal volumes were assessed via OCT and HFUS.

### *In vivo* experiments

OCT and HFUS measurements were performed using 21 BALB/c mice. Before imaging, the animals were anesthetized via intraperitoneal injection of xylazine (10 mg/kg) and ketamine (80–100 mg/kg). The animals’ fur was depilated at the dorsum (Veet crème sensitive, Reckitt Benckiser) and the area subsequently disinfected (Isozid-H, Gebro Pharma). Metamizol (100 mg/kg) was given for analgesia. Four hydrogel deposits per mouse, each containing 10 μl of the filler, were injected into the dorsal dermis using a 250 µl microliter syringe (Hamilton) and a 27 gauge needle (n = 84). Photographs (Canon PowerShot G15, Canon Deutschland GmbH) of the dorsum of each mouse were taken. At the end of the experiment, animals were sacrificed and the respective skin areas were stained with hematoxylin and eosin for histological evaluation. The experimental protocols were approved by the local Animal Welfare Committee of the Medical University of Vienna and the Federal Ministry of Science, Research and Economy (GZ 66.009/0380-WF/V/3b/2016, GZ BMWFW-66.009/0299-WF/V/3b/2017). All experiments were performed in full accordance with all relevant regulations and the ARRIVE guidelines^33^.

### Volumetric segmentation

In order to obtain three-dimensional representations of the applied deposits and to measure their respective intradermal volumes, OCT and HFUS data sets were first used to develop a machine-learning-based algorithm for automatic segmentation^31^. Based on this algorithm, filler borders within the generated cross-sectional images were automatically segmented and the volume was calculated, taking into account the scan range in both lateral dimensions and the refractive indices of the media.

### Statistical analysis

Statistical analysis was performed in SPSS (IBM SPSS Statistics, Version 25). To compare means of volumetric data, paired t-tests were used. A Pearson correlation was performed for HFUS and OCT values. For all evaluations, a significance level of α = 0.05 was applied. Finally, a Bland-Altman plot was used to compare OCT and HFUS data.

## Data Availability

Data generated in the present study are available from the corresponding author upon reasonable request.
